# Common Mental Disorders, Functional Limitation and Diet Quality Trends and Related Factors among COPD Patients in Spain, 2006–2017: Evidence from Spanish National Health Surveys

**DOI:** 10.3390/jcm10112291

**Published:** 2021-05-25

**Authors:** Silvia Portero de la Cruz, Jesús Cebrino

**Affiliations:** 1 Department of Nursing, Pharmacology and Physiotherapy, Faculty of Medicine and Nursing, University of Córdoba, Avda. Menéndez Pidal, S/N, 14071 Córdoba, Spain; n92pocrs@uco.es; 2 Department of Preventive Medicine and Public Health, Faculty of Medicine, University of Seville, Avda. Doctor Fedriani, S/N, 41009 Seville, Spain

**Keywords:** activities of daily living, diet, mental disorders, pulmonary disease, chronic obstructive

## Abstract

Certain conditions such as common mental disorders (CMDs), functional limitation (FL) and poor diet quality may affect the lives of individuals who suffer from chronic obstructive pulmonary disease (COPD). This study sought to examine time trends in the prevalence of CMDs, FL and diet quality among male and female COPD patients living in Spain from 2006 to 2017 and to identify which factors were related to CMDs, FL and a poor/improvable diet quality in these patients. We performed a cross-sectional study among COPD patients aged ≥ 40 years old using data from the Spanish National Health Surveys conducted in 2006, 2011 and 2017, identifying a total of 2572 COPD patients. Binary logistic regressions were performed to determine the characteristics related to CMDs, FL and poor/improvable diet quality. Over the years of the study, the prevalence of FL among female COPD patients increased (*p* for trend <0.001). In addition, CMDs were associated to body mass index (BMI), educational level, physical activity, smoking status, occupation, chronic conditions and alcohol consumption; FL was related to age, living with a partner, educational level, physical activity and chronic conditions; and poor/improvable diet quality was associated to age, smoking status, BMI and physical activity.

## 1. Introduction

Chronic obstructive pulmonary disease (COPD) is a highly significant public health problem affecting around 174 million of adults aged 40 years and over, with high morbidity and mortality [1]. According to World Health Organization, COPD is the third leading cause of death [2]. Moreover, this chronic condition results in a high economic burden: globally, previous studies report that the direct cost of management of COPD ranges from 61% to 89% of the total cost of health care [3,4].

COPD patients typically have several comorbidities, including heart failure, skeletal muscle dysfunction, osteoporosis and mental health issues, such as anxiety and depression [5]. The importance of the psychiatric comorbidity in COPD has recently been stressed, and it has been suggested that common mental disorders (CMDs) in COPD patients are associated with sleep disorders, lacking interests, asthenia, lower levels of self-efficacy, more frequent exacerbations and more hospital admissions [6,7]. These have a serious impact on the patients’ quality of life, coping strategies and adherence to treatment [8,9], contributing to the overall disease burden of COPD [10]. The prevalence of depression and anxiety in COPD patients is greater compared to the general population, ranging from 9.8% to 57% in the case of depression [11,12,13,14] and from 8.1% to 46% for anxiety [11,12,15], although both of these mental disorders are underdiagnosed [16]. Nevertheless, it should be noted that depressive and anxiety symptoms are both correlated to pulmonary-specific symptoms among patients with COPD, potentially leading to overestimate or misdiagnosis of depression and anxiety, which can be avoided with a robust clinical interview [17]. Recent studies have highlighted the influence of age, educational status, smoking habits and alcohol consumption on CMDs in COPD sufferers [18,19]. However, the role of other characteristics remains controversial.

COPD significantly affects peoples’ lives, leading to an inability to finish meaningful activities [20]. As COPD progresses, individuals experience numerous symptoms, of which dyspnea, fatigue and muscle weakness are the most severe [17,21]. This consequently affects their ability to complete both daily life activities, such as dressing, stair climbing and household chores, and more complex tasks known as instrumental activities of daily living, such as shopping, housework and using public transport and individuals suffering from COPD face a difficult process of adaptation to their health condition [22]. Even though a number of studies have pointed out that the most widely recognized conditions causing functional limitations among people are rheumatoid arthritis, spine issues and cardiovascular disease and that these conditions increase in prevalence as the population ages [23,24], Djibo et al. [25] reported that the highest rates of functional limitation occur in middle-aged adults (45–64 years old) with COPD, although the reasons for this finding are not completely understood. In fact, patterns of functional limitation in activities of daily living and related determinants, specifically in COPD patients, are currently under investigation [26]. 

Even though cigarette smoking is one of the most important risk factors for development and progression in COPD [27], the symptoms advance even after the patient has quit smoking [28]. Hence, there are probably additional modifiable elements which contribute to COPD, and these need to be recognized in order to improve patient management. Diet is one of the most important lifestyle determinants of health; however, relatively little attention is paid to diet in relation to lung problems and the current dietary guidelines do not provide enough information in this regard [29]. Evidence available from previous studies suggests that diet is an important contributor to the symptoms, functional limitation and prognosis of COPD [30,31]. 

Oxidative stress and associated inflammation are thought to be involved in COPD development and progression [32,33]. Diet may contribute to antioxidant/oxidant and inflammatory status in COPD. Previous studies have reported the potential beneficial effects of foods rich in antioxidants (fruits and vegetables) on the reduction of COPD symptoms and incidence [30,34,35]. In addition, a protective effect against development of COPD has been suggested for fish consumption. Fish oils are thought to have anti-inflammatory effects, because of the influence of the n-3 polyunsaturated fatty acids eicosapentaenoic acid and docosahexaenoic acid on arachidonic acid metabolism [36]. Moreover, among micronutrients, it has been found a positive association between intake of calcium, phosphorus, iron, potassium and selenium and lung function [36]. Other studies support a role for vitamin D in preventing and reducing the number of COPD exacerbation [37,38]. Additionally, important diet related factors such as loss of fat free mass [39] and obesity [40] are relevant to prognosis, functional limitation and COPD symptoms. Nevertheless, COPD patients show an inadequate diet quality, consuming less fruit and vegetables and more foods with a lower antioxidant content, compared to a healthy population [34], which is considered the major cause of malnutrition in these people. Nowadays, evidence showing how sociodemographic and health-related factors contribute to diet quality in this population is scarce. 

Previous evidence has showed that COPD is expressed differently in women than in men in relation to symptoms, pulmonary function, exacerbations or comorbidities [41] and there is therefore a need to clarify the role of gender in this illness, in order to optimize the care and attention of these patients. 

Finally, it should be noted that CMDs can be a cause and a consequence of COPD and diet is associated with inflammation, oxidative stress and brain function (which are involved in developing CMDs but also may cause impact on COPD disease and progression). All of these factors are relevant to functional limitation of people. Therefore, the sociodemographic and health-related behaviors variation over time can impact on prevalence of CMDs, functional limitation and diet quality among individuals with COPD [42]. To the best of our knowledge, this study is the first in COPD patients living in Spain to measure and analyze the presence of CMDs, functional limitations, diet quality and gender-related factors at the same time. This will provide us with vital data to plan more personalized and efficient care programs to deal with COPD. Therefore, the main objectives of the present study were to examine time trends in the prevalence of CMDs, functional limitation and diet quality among male and female COPD patients living in Spain from 2006 to 2017, and to identify which characteristics, including sociodemographic, health-related variables and the use of clinical preventive care services, were related to CMDs, functional limitation and a poor/improvable diet quality among men and women suffering from COPD.

## 2. Materials and Methods

### 2.1. Design, Data Source and Participants

The data for this nationwide cross-sectional study were obtained from the personalized interviews of the Spanish National Health Survey (SNHS) 2006 [43], the SNHS 2011 [44] and the SNHS 2017 [45]. The interviews were conducted with community people (non-institutionalized) residing in main family dwellings (household) of Spain by the Ministry of Health, Consumer Affairs and Social Welfare in partnership with the National Institute of Statistics. A three-stage probabilistic design was used, stratifying the data by census section, households and persons. The individuals were informed about the survey by letter, specifying the reasons for the survey and the voluntary and anonymous nature of participation, and about the visit of a suitably approved interviewer. 

All individuals aged ≥40 years old were considered. Those who responded “yes” to the query, “Has your doctor told you that you are currently suffering from chronic bronchitis, emphysema or COPD?” were identified as suffering from COPD. The sample initially comprised of 3373 participants (SNSH 2006: *n* = 1326; SNSH 2011: *n* = 1002; SNHS 2017: *n* = 1045), although 801 (23.8%) subjects were later excluded due to non-response or refusal to respond to the questions (SNSH 2006: *n* = 406; SNSH 2011: *n* = 212; SNHS 2017: *n* = 183), although their characteristics were similar to the other COPD patients included. Finally, the total sample consisted of 2572 subjects: 920 in SNHS 2006; 790 in SNHS 2011; and 862 in SNHS 2017.

### 2.2. Outcome Measurements

CMDs. CMDs are defined as minor psychiatric disturbances with a set of non-psychotic depressive, and anxiety symptoms that affect the performance of daily-life activities. They included symptoms such as fatigue, insomnia, memory lapses, difficulty of concentration and irritability [46]. These were identified using the 12-item General Health Questionnaire (GHQ-12) [47] in Spain [48,49]. The GHQ-12 detects mainly two types of disorders: anxiety and depression. The questionnaire consists of 12 Likert-type items with four possible answers from 0 (less than usual) to 3 (much more than usual). It is assigned 0 points to answers 0 and 1, and 1 point to answers 2 and 3 [50]. The overall score is the sum of the points of the 12 items, thus, the total score ranges between 0 and 12 points. A cut-off point at ≥3 points was considered, so that the absence of CMDs was determined by scores <3 points and their existence denoted by scores ≥3 points, the latter being used to indicate the risk of psychological distress [51].

Functional limitation. This variable was examined by considering questions focusing on different physical tasks in two functional areas [52,53]: (i) basic activities of daily life, which require managing one’s basic physical needs (e.g., personal hygiene, dressing, toileting, transferring or ambulating); (ii) instrumental activities of daily life, which include more complex activities related to the ability to live independently in the community (e.g., food preparation, managing finances and medications or housekeeping). The level of difficulty for all items was classified as follows: “I can do it by myself”, “I can do it with someone’s help” or “I am unable to do it at all”. We defined the existence of functional limitation when subjects answered “I can do it with someone’s help” or “I am unable to do it at all” on at least one item for a specific area of functional limitation.

Diet quality. This was evaluated using the Spanish Health Eating Index (SHEI) [54], which is based on the North American Healthy Eating Index [55]. The SHEI includes ten food groups (cereals, vegetables, fruit, dairy, meat, legumes, cold meats, sweets, soft drinks and dietary variety) divided into five categories (never or hardly ever, <once per week, 1–2 times per week, ≥3 times per week, but not daily, and daily), considering the Spanish recommendations [56]. The scores referring to each food group ranged from 0 to 10 points, as shown in Supplementary Table S1. The total score is obtained by adding the frequency of consumption of the different food groups. The higher the score, the higher the level of agreement with the Spanish recommendations. The overall SHEI score was classified taking into account the following cut-offs described previously [55]: >80 points, good diet quality; 51–80 points, improvable diet quality; and <51 points, poor diet quality.

### 2.3. Sociodemographic Variables, Health-Related Determinants and Clinical Preventive Care Services

The following data were recorded:

Sociodemographic variables: Year of the surveys (2006, 2011, 2017); gender (women, men); age group (40–54 years, 55–69 years, ≥70 years); educational attainment (without studies, primary, secondary or professional training, university); town population (<10,000 inhabitants, 10,000–100,000 inhabitants, >100,000 inhabitants); living with a partner (yes, no) and occupation (working, non-working).

Health-related determinants: Body Mass Index (BMI), which was categorized according to the cut-offs proposed by the World Health Organization [57] (underweight, normal weight, overweight and obese); smoking status (current smokers, ex-smokers or non-smokers); alcohol intake in the last year (yes, no); leisure-time physical activity (yes, no) and number of medical diagnosed of comorbid chronic conditions (myocardial infarction, diabetes, chronic heart disease, arthritis, high blood pressure, asthma, cancer and stroke), classified as none, one or two and three or more. 

Clinical preventive care services: Blood cholesterol level measurement in the last year (yes, no); blood pressure taken by a healthcare professional in the last year (yes, no) and influenza vaccination during the last vaccination campaign (yes, no).

### 2.4. Procedure and Ethical Considerations

The anonymized information downloaded is accessible to the general population through the National Institute of Statistics and Ministry of Health, Consumer Affairs and Social Welfare sites [43,44,45]. As indicated by Spanish law, Ethics Committee endorsement is not required if secondary information is being used. The research data is included in the Supplementary File.

### 2.5. Statistical Analysis

Descriptive analysis was carried out using counts, percentages and mean and standard deviation. Inferential analysis was performed using Chi-square test or Fisher’s exact test if the number of expected frequencies was greater than 5. Chi-square trend analysis was also performed for the presence of CMDs, physical limitation and poor/improvable diet quality taking into account the years of the study. For each dependent variable (CMD, functional limitation and diet quality) we fitted multivariate logistic regression models. We included all the variables whose univariate test showed potential association with each dependent variable (*p* ≤ 0.15), and backward selection was used to eliminate non-significant variables based on the probability of the Wald statistic. Crude and adjusted Odds Ratios (OR) were calculated with 95% confidence intervals. The goodness of fit was verified with the Hosmer–Lemeshow test. A *p*-value ≤ 0.05 was considered to be significant. All data analyses were performed separately for women and men. The statistical analysis was carried out using the IBM SPSS Statistical package version 25 (IBM Corp, Armonk, NY, USA), licensed to the University of Cordoba (Spain).

## 3. Results

### 3.1. Sociodemographic Variables, Health-Related Determinants and Use of Clinical Preventive Care Services

The total sample was made up of 2572 individuals with COPD, including 50.4% (*n* = 1295) women and 49.7% (*n* = 1277) men. The groups did not differ in some variables, including level of education, occupation, number of chronic diseases, measurement of blood pressure and the amount of total serum cholesterol in the last 12 months and Influenza vaccination during the last campaign. In contrast, the men were significantly older (*p* < 0.01), were more likely to be classified as overweight (*p* < 0.01) and reported more frequently doing physical activity during their leisure time (*p* < 0.01). The women reported more frequently living in towns with >100,000 inhabitants (*p* < 0.01) and not consuming tobacco (*p* < 0.01) or alcohol in the last 12 months (*p* < 0.01) (Table 1).

### 3.2. CMDs, Functional Limitation and Diet Quality

**Figure 1:**
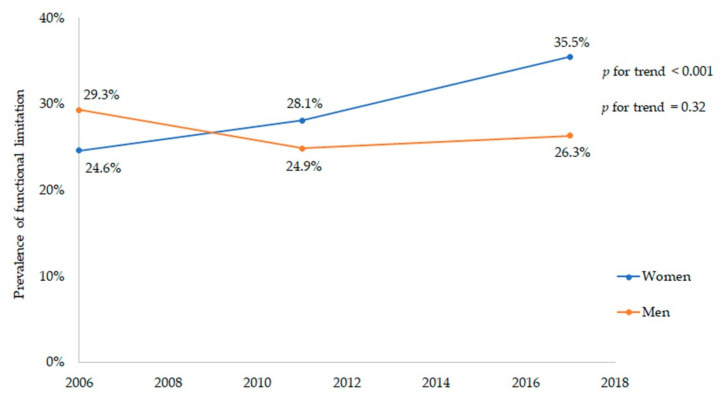
Prevalence of functional limitation in COPD patients over time (*n* = 2572).

The women suffered from CMDs more frequently than men (45.5% vs. 30.2%, respectively; *p* < 0.001). However, the men reported more frequently having poor/improvable diet quality than the women (61.3% vs. 53.1%, respectively; *p* < 0.001). Regarding functional limitation, we found no differences between both groups. Nevertheless, over the study years, the prevalence of functional limitation among women with COPD increased (*p* for trend < 0.001) (Figure 1) but not for men (*p* for trend = 0.32). No significant changes were observed in either men or women over the study period in relation to CMDs (men in 2006: 31.1%, in 2011: 30.3%, in 2017: 29.2%, *p* for trend = 0.55; women in 2006: 46.8%, in 2011: 47.4%, in 2017: 42.4%, *p* for trend = 0.17) and poor/improvable quality of diet (men in 2006: 63.7%, in 2011: 52.2%, in 2017: 67.6%, *p* for trend = 0.19; women in 2006: 43.8%, in 2011: 56.8%, in 2017: 41.7%, *p* for trend = 0.41).

Table 2 shows the distribution of CMDs, functional limitation and poor/improvable diet quality according to the study variables from 2006 to 2017 in men with COPD. We observed an increase in the prevalence of CMDs from 2006 to 2017 in workers (2006: 17.1%, 2011: 23.2%, 2017: 31.9%, *p* < 0.001). In addition, we found an increase in the prevalence of functional limitation in those who had ≥3 chronic conditions (2006: 45.2%, 2011: 38.5%, 2017: 48.6%, *p* < 0.001). In relation to poor/improvable diet quality, this variable decreased over the study years in individuals who had university studies (2006: 64.9%, 2011: 44.8%, 2017: 63.0%, *p* < 0.001).

When comparing the results of the three surveys in women with COPD (Table 3), we found an increase in the prevalence of functional limitation over the study years in those who were obese (2006: 34.3%, 2011: 36.7%, 2017: 41.0%, *p* < 0.001) and had ≥3 chronic conditions (2006: 39.3%, 2011: 49.7%, 2017: 57.4%, *p* < 0.001). Regarding poor/improvable diet quality, this variable decreased over the study years in those who had university studies (2006: 55.6%, 2011: 44.0%, 2017: 51.4%, *p* < 0.001).

### 3.3. Association between Sociodemographic Variables, Health-Related Determinants, Use of Clinical Preventive Care Services and CMDs, Functional Limitation and Diet Quality

CMDs, functional limitation and diet quality were distributed differently according to the sociodemographic factors, health-related variables and use of clinical preventive care services in men with COPD (Table 4). In the adjusted multivariate analysis, the probability of CMDs was lower among those who were normal weight, had university studies, took part in physical activity in their leisure time and who had no or 1–2 chronic conditions, and were higher among those who had consumed alcohol during the last 12 months. In addition, having primary, secondary and university studies, participating in physical activity during leisure time and having no or 1–2 chronic conditions was associated with a lower probability of functional limitation. In relation to diet quality, being between 40–54 years old and being a smoker were associated with a higher probability of poor/improvable diet quality in men with COPD, while taking part in physical activity during leisure time decreased that probability. 

Table 5 shows the variables associated with the presence of CMDs, functional limitation and poor/improvable diet quality in women with COPD. In the adjusted multivariate analysis, the probability of CMDs was higher in those who were smokers and had consumed alcohol in the last year and lower in those who were workers and had no or 1–2 chronic conditions. Additionally, being between 55–69 years old, having primary, secondary or university studies, living with a partner, participating in physical activity during leisure time and having no or 1–2 chronic conditions were variables associated with a lower probability of functional limitation in women with COPD. Regarding diet quality, being between 40–54 years old, being underweight and being a smoker were associated with a higher probability of poor/improvable diet quality in women with COPD.

## 4. Discussion

### 4.1. Main Findings Related to Sociodemographic Variables, Health-Related Determinants and Use of Clinical Preventive Care Services

The SNHS has been used previously due to its statistical potential as a representative national survey with a substantial sample size. According to previous evidence, the results of the current study showed that, in comparison to men, women with COPD were younger, smoked less, were more frequently obese, lived alone in towns with a greater number of inhabitants and took part in less physical activity [58,59,60,61,62,63].

### 4.2. Main Findings Related to the Presence of a CMD

In our study, although women suffered from a CMD more frequently than men, probably due to their tendency to internalize emotions [13], the prevalence of CMDs increased over time among male workers with COPD. There are complex associations between COPD, its symptoms, psychological impacts, health-related behaviors and work productivity. In that sense, the relationships between anxiety/depression and COPD outcomes is thought to be cyclical: elevated level of anxiety/depression symptoms may increase negative health-related behaviors, such as smoking, which, in turn, exacerbate COPD symptoms being able then further exacerbate anxiety and depression symptoms [64] having a direct negative effect on work productivity [65]. On the other hand, alcohol consumption was related to a greater likelihood of suffering from CMDs in women and men, whereas having none, one or two chronic conditions made it less likely. While several studies have found an association between drinking alcohol and COPD and many individuals with COPD have been or are heavy drinkers [66,67], there is little research into how alcohol use impacts on the mental status of people living with COPD [68]. Considering the biopsychosocial effects of alcohol use in COPD patients, it therefore seems important to design strategies to reduce their alcohol consumption by gender. Especially, taking into account that, in the current study, men reported more frequently consuming alcohol in the last 12 months compared to women. Moreover, future research should explore longitudinally whether alcohol consumption is used as a coping strategy with mental disorders, or it is a manifestation of mental distress. Regarding chronic conditions, the mechanisms that help to explain the high frequency of major comorbidities in individuals with COPD focus on the concept of systemic inflammation in addition to coexisting conditions that one would naturally expect (e.g., due to the patients’ advanced age) and due to share risk factors [69]. The link between COPD and mental disorders such as anxiety and depression may be systemic inflammation due to spillover [69]. The lower probability of CMDs in COPD subjects with no or fewer chronic conditions found in the current study could be explained by the fact that these COPD participants are less likely to be functionally limited, making them less vulnerable to social isolation, social interaction and dependency on caregivers. 

In addition, in our study, being a worker was associated with a lower probability of CMDs in women. In this context, however, the findings from the literature are unclear and contradictory. Some authors suggest that employment is beneficial for psychological well-being because it temporarily relieves a woman of the stress arising from gender role expectations, especially if the woman works full-time and has no children [70]. Our study demonstrated that physical activity decreased the probability of suffering from CMDs in men with COPD. It has also been reported that men with COPD take part in significantly more physical activity than women and that physical activity relieves their symptoms, improves their quality of life and decreases the risk of exacerbations [13]. Additionally, in the current study, being a smoker was related to an increased likelihood of CMDs in women, which supports previous reports [60,71]. Female COPD patients are more likely to suffer from depression and anxiety than their male counterparts, with manifestations that can be severe [60]. It has been suggested that the relationship between smoking and depression/anxiety may be bidirectional, with smoking used to alleviate symptoms and these symptoms in turn leading to further smoking [72]. 

In the current study, men with a high educational level had less probability of suffering from a CMD than men without studies, which is confirmed by international research which shows that a greater level of education is related to a better mental health [73]. One factor that should be considered is that CMDs are less likely to occur in men than women with COPD [15], and men with a high level of education are likely to understand their own disease better and implement self-management of COPD, which could contribute to better mental health [74]. Few studies have evaluated the relationship between BMI and mental problems in individuals with COPD and the findings which do exist are noticeably contradictory. While a number of studies noted that a lower BMI is significantly related to the development of mental issues in COPD patients [75,76], other authors suggest positive associations between weight and mental health problems in COPD patients [40]. In our study, we found that normal-weight men had less probability of suffering from CMDs, which matched results from another study [74]. Therefore, in COPD individuals, it has been suggested that BMI levels <21.38 kg/m^2^ could help to identify COPD individuals with a greater risk of depression [74].

### 4.3. Main Findings Related to Functional Limitation

The present study reveals that the prevalence of functional limitation increased from 2006 to 2017 in women with COPD but not in men. Numerous studies have suggested that functional limitation seems to decrease [77,78] or increase [79] over time. Previous studies have reported some factors that might be responsible for those changes in time in individuals with COPD. Thus, living in a disadvantaged neighborhood is associated with worse COPD-related outcomes, including functional limitations [80]. The available evidence has also shown that different personalities, resilience or coping styles, as well as paid work and a decline in economic conditions could help to explain the variation in the prevalence of functional limitation over the years [20,81]. Additionally, it seems that having a pet can offer both psychological and physical health benefit decreasing both sedentary lifestyle and levels of functional limitation [82]. In women with COPD, one of the reasons behind this could be the fact that women’s health is often ignored due to gender disparity; also, females with COPD experience a greater risk of exacerbation and more severe symptoms than men, producing severe stages of COPD and generating a higher prevalence of functional limitation [25,60]. In the current study, the prevalence of functional limitation increased over time in obese women with COPD. This indicates the relevance of devising interventions to reduce functional limitation, including adaptation of the environment and studying, at the beginning of the intervention, the activities in which these obese individuals experience a higher degree of functional limitation.

Factors associated with a lower probability of suffering from functional limitation in both females and males included higher educational attainment, performing physical activity during leisure time and having no, one or two chronic diseases. Higher-educated individuals may be able to marshal their economic and psychosocial resources to avoid functional limitation, premature death and disease, even in circumstances not conducive to health [83]. In this regard, Assari et al. [84] observed a protective effect of a higher educational level against COPD. As regards performing physical activity during leisure time, previous research has indicated that leisure activities are among the first activities to be given up among individuals with COPD, as they are not perceived as having as much importance as other activities in life [85]. In addition, associations between functional limitation and COPD may be influenced by comorbidities. This result is in line with those from previous studies that showed that the number of incident chronic diseases is associated with a loss of functional independence [24,86,87]. Our data showed that the prevalence of functional limitation increased over time among men and women who suffered from COPD and had ≥3 chronic conditions. As dependence in basic and instrumental activities of daily life may indicate an early sign of functional decline, intervention programs targeting major modifiable comorbidities for COPD might help to reduce the risk of functional limitation and its progression in these individuals.

Another factor that can influence functional limitation is age. In our study, being between 55–69 years old decreased the probability of functional limitation in women, but not in men. Previous studies show that there is a higher prevalence of arthritis/musculoskeletal symptoms in COPD individuals, especially in women [88,89] and that functional limitation among those ages is most often attributed to these problems [90]. Despite the fact that persons with disabilities generally experience greater barriers in accessing healthcare services than the general population [91], women overall have a higher level of health-care utilization, make more primary care visits and receive more diagnostic services than men [92], which could result in a lower probability of functional limitation in women within that age group.

Our findings showed that 64.09% of women with COPD lived alone. While some studies point to a variation from 16.30% [93] to 40% [40], the higher prevalence obtained in our study can be due to differences in the aims of the study. Nonetheless, women with COPD who lived with a partner were less likely to have functional limitation. Even though women are cared for less frequently by their partners [40], the partners of COPD patients are an essential factor in helping them to cope with the disease, making them less dependent for daily activities [94]. An early assessment of social status would provide the opportunity to recognize COPD individuals in greater need of social support and COPD care programs given by formal or informal caregivers. 

### 4.4. Main Findings Related to Diet Quality

In our analysis, men had a worse diet quality than women. Here, it is crucial to consider the potential gender differences which may influence the different food choices [95]. Moreover, our results showed that the prevalence of poor/improvable diet quality decreased from 2006 to 2017 among men and women with higher levels of education, probably due to the fact that better-educated individuals generally have a greater ability to cook and comprehend dietary guidelines, as well as a higher capacity to understand nutritional interventions [36]. Additionally, the findings of the current study showed that the likelihood of poor/improvable diet quality was greater in individuals who were between 40–54 years old and were smokers in both genders. In relation to age, these findings could be explained by people’s tendency to improve their diet as they get older, as they look after themselves more, which reduces the impact exerted by early-life and cumulative dietary choices on later-life COPD development and progression [36]. On the other hand, in the general population, smokers have significantly lower diet quality scores in relation to non-smokers [96], which was consistent with our results. In this context, it has been reported that tobacco smoke may lead to the dysregulation of appetite and changes in the sense of taste, which may lead smokers to choose certain foods over others [97]. Generally, the dietary habits of smokers are defined by higher intakes of energy, saturated fat, cholesterol and alcohol and by lower intakes of antioxidant vitamins and fiber [98]. This suggests that some of these differences may aggravate the harmful effects of the components in tobacco smoke on COPD [99]. 

For underweight women with COPD, the likelihood of poor/improvable diet quality was greater. Women with COPD have a higher prevalence of low BMI than their male counterparts [59]. Indeed, previous studies have shown that COPD subjects with low BMI have a shorter period of survival [99] and a greater risk of acute exacerbations with worse airflow limitation, higher level of inflammation biomarkers and poorer health status [100]. 

In the current study, male COPD patients who took part in physical activity had a lower likelihood of poor/improvable diet quality. A recent study carried out in Spanish individuals with COPD reported that males are more likely to be involved in physical activity [101]. Being physically active reduces the risk of hospitalization and daily symptoms of dyspnea and fatigue, improves quality of life and predicts an increase in healthy dietary patterns [102,103,104].

### 4.5. Strengths and Limitations

The present study has certain limitations. First, since it was a cross-sectional study, the causality cannot be demonstrated. Second, the definition of COPD used was based on self-reported physician-diagnosis, although it must be mentioned that this definition has been commonly used in large national and international surveys [13,84,105]. Third, disease severity, such as lung function or exacerbations, was not measured and psychological symptoms and limitations are both related to disease severity so that the interpretation of the data should be done with caution. Fourth, the use of the SHEI to assess the diet quality did not show the frequency of consumption by quantities of food or energy; however, the validity of the Health Eating Index (HEI) has been demonstrated in studies with plasma biomarkers [106,107]. Fifth, the use of yes/no responses on alcohol intake and leisure activity can result in loss of granularity due to does not allows understanding of the harm from hazardous alcohol consumption or discriminate in terms of healthy behaviors. Sixth, BMI was calculated from the heights and weights reported by the subjects, which may not be accurate. Finally, due to the fact that data from SNHS is stored anonymously, it is impossible to know if a participant has taken part in more than one survey. On the other hand, the strength of the current study includes the use of national standardized representative surveys with a large sample size.

### 4.6. Implications for Research and Practice

The findings of the current study have a number of potential implications for research and clinical practice. Firstly, as regards diet quality, our findings require confirmation in prospective cohorts, but suggest the need to consider gender differences in the selected sociodemographic characteristics and lifestyle factors in order to optimize the management of COPD patients by designing suitable dietary guidelines for a diet which is beneficial for lung health. Secondly, CMDs should be assessed in routine care and affective factors should be properly monitored in COPD patients. Additionally, further interventions based on cognitive behavioral therapy are needed, as they appear to be effective in improving CMDs in COPD sufferers [108]. This could be accomplished by using self-report measures as part of the regular screening in both primary care and pulmonary clinics. Finally, measuring functional limitation in people with COPD in addition to their sociodemographic and lifestyle characteristics may reveal how functional limitations affect an individual’s independence and generate information to help us design more personalized and efficient care programs. Given the diverse membership of the health care teams involved in the care of individuals living with COPD, there is a need for collaborative interdisciplinary work to engage the care workers as active participants in their care and in the decision-making processes related to their health.

## 5. Conclusions

This study shows an increased prevalence of functional limitation among women with COPD over time. Moreover, we observed that the number of men with COPD who suffered from CMDs increased from 2006 to 2017. In the same way, the prevalence of functional limitation increased among male and female COPD patients who had ≥3 chronic conditions and in female COPD patients who were obese. Conversely, the prevalence of poor/improvable diet quality decreased over time in men and women with COPD who had university studies. In men, CMDs were associated with BMI, educational level, physical activity, chronic conditions and alcohol consumption and the presence of functional limitation was related to educational level, physical activity and chronic conditions. In addition, poor/improvable diet quality was associated to age, smoking status and physical activity. In women, CMDs were linked with BMI, educational level, smoking status, occupation, alcohol consumption and chronic disease, and the presence of functional limitation was associated with age, educational level, living with a partner, physical activity and chronic conditions. Finally, poor/improvable diet quality was associated with age, BMI and smoking status.

## Figures and Tables

**Table 1:** Comparison of male and female COPD patients as regards sociodemographic variables, health-related determinants and the use of clinical preventive care services (*n* = 2572).

Variables	Total *n* (%)	Men *n* = 1277 (%)	Women *n* = 1295 (%)	*p*-Value
Educational attainment				0.05
Without studies	707 (27.5)	320 (25.0)	387 (29.9)	
Primary	823 (32.0)	423 (33.1)	400 (30.9)	
Secondary or PT	832 (32.3)	422 (33.1)	410 (31.6)	
University	210 (8.2)	112 (8.8)	98 (7.6)	
Town population				<0.01
<10,000 inhabitants	687 (26.7)	382 (29.9)	305 (23.5)	
10,000–100,000 inhabitants	850 (33.1)	414 (32.4)	436 (33.7)	
>100,000 inhabitants	1035 (40.2)	481 (37.7)	554 (42.8)	
Living with a partner				<0.01
Yes	1127 (43.8)	662 (51.8)	465 (35.9)	
No	1445 (56.2)	615 (48.2)	830 (64.1)	
Occupation				0.67
Working	423 (16.5)	214 (16.8)	209 (16.1)	
Non-working	2149 (83.5)	1063 (83.2)	1086 (83.9)	
Body Mass Index				<0.01
Underweight	34 (1.3)	17 (1.3)	17 (1.3)	
Normal weight	720 (28.0)	331 (25.9)	389 (30.0)	
Overweight	1000 (38.9)	565 (44.3)	435 (33.6)	
Obesity	818 (31.8)	364 (28.5)	454 (35.1)	
Smoking status				<0.01
Smoker	578 (22.5)	332 (26.0)	246 (19.0)	
Ex-smoker	888 (34.5)	695 (54.4)	193 (14.9)	
Non-smoker	1106 (43.0)	250 (19.6)	856 (66.1)	
Alcohol intake in the last year				<0.01
Yes	676 (26.3)	371 (29.1)	305 (23.6)	
No	1896 (73.7)	906 (70.9)	990 (76.4)	
Leisure-time physical activity				<0.01
Yes	1364 (53.0)	716 (56.1)	648 (50.0)	
No	1208 (47.00)	561 (43.9)	647 (50.0)	
Number of chronic conditions				0.17
0	367 (14.2)	216 (16.9)	151 (11.7)	
1–2	1388 (54.0)	708 (55.5)	680 (52.5)	
≥3	817 (31.8)	353 (27.6)	464 (35.8)	
Measurement of blood pressure in the last 12 months				0.71
Yes	227 (8.8)	1167 (91.4)	1178 (91.0)	
No	2345 (91.2)	110 (8.6)	117 (9.0)	
Measurement of the amount of total serum cholesterol in the last 12 months				0.94
Yes	2204 (85.7)	1095 (85.8)	1109 (85.6)	
No	368 (14.3)	182 (14.2)	186 (14.4)	
Influenza vaccination during the last campaign				0.72
Yes	1481 (57.6)	786 (61.6)	695 (53.7)	
No	1091 (42.4)	491 (38.4)	600 (46.3)	
	**Mean (SD)**	**Mean (SD)**	**Mean (SD)**	***p*-value**
Age (years old)	67.0 (12.8)	67.8 (12.4)	66.2 (13.1)	<0.01

SD: Standard Deviation; PT: professional training.

**Table 2:** Distribution of the CMDs, functional limitation and poor/improvable diet quality according to the study variables in men with COPD from 2006 to 2017 (*n* = 1277).

	Common Mental Disorders			Functional Limitation			Poor/Improvable Diet Quality		
	*n* = 386			*n* = 344			*n* = 783		
**Variables**	2006	2011	2017	2006	2011	2017	2006	2011	2017
	*n* = 142 (%)	*n* = 123 (%)	*n* = 121 (%)	*n* = 134 (%)	*n* = 101 (%)	*n* = 109 (%)	*n* = 291 (%)	*n* = 272 (%)	*n* = 280 (%)
Age group									
40–54	15 (25.0)	27 (36.0)	28 (38.4)	-	-	-	48 (80.0)	54 (72.0)	62 (84.9)
55–69	45 (26.9)	42 (29.0)	37 (25.7)	17 (10.2)	18 (12.4)	13 (9.0)	103 (61.7)	76 (52.4)	97 (67.4)
≥70	82 (35.7)	54 (29.0)	56 (28.4)	117 (50.9)	83 (44.6)	96 (48.7)	140 (60.9)	82 (44.1)	121 (61.4)
Educational attainment									
Without studies	47 (39.5)	32 (27.1)	32 (38.6)	50 (42.0)	45 (38.1)	39 (47.0)	67 (56.3)	52 (44.1)	56 (67.5)
Primary	62 (28.7)	28 (32.6)	35 (28.9)	65 (30.1)	18 (20.9)	43 (35.5)	142 (65.7)	40 (46.5)	82 (67.8)
Secondary or PT	26 (30.6)	60 (34.7)	43 (26.2)	10 (11.8)	32 (18.5)	17 (10.3)	58 (68.2)	107 (61.9)	113 (68.9)
University	7 (18.9)	3 (10.3)	11 (23.9)	9 (24.3)	6 (20.7)	10 (21.7)	24 (64.9) ^1^	13 (44.8)	29 (63.0)
Town population									
<10,000 inh	43 (28.7)	31 (27.2)	30 (25.4)	44 (29.3)	29 (25.4)	35 (29.7)	100 (66.7)	64 (56.1)	82 (69.5)
10,000–100,000 inh	52 (35.1)	45 (36.9)	50 (34.7)	49 (33.1)	29 (23.8)	44 (30.6)	86 (58.1)	63 (51.6)	95 (66.0)
>100,000 inh	47 (29.6)	47 (27.7)	41 (27.0)	41 (25.8)	43 (25.3)	30 (19.7)	105 (66.0)	85 (50.0)	103 (67.8)
Living with a partner									
Yes	91 (29.9)	39 (22.7)	48 (25.8)	84 (27.6)	48 (27.9)	52 (28.0)	184 (60.5)	86 (50.0)	111 (59.7)
No	51 (33.3)	84 (35.9)	73 (32.0)	50 (32.7)	53 (22.7)	57 (25.0)	107 (69.9)	126 (53.9)	169 (74.1)
Occupation									
Working	13 (17.1) ^1^	16 (23.2)	22 (31.9)	-	-	-	59 (77.6)	45 (65.2)	59 (85.5)
Non-working	129 (33.9)	107 (31.8)	99 (28.7)	134 (35.2)	101 (30.0)	109 (31.6)	232 (60.9)	167 (49.6)	221 (64.1)
Body Mass Index									
Underweight	-	2 (33.3)	7 (63.6)	-	2 (33.3)	4 (36.4)	-	3 (50.0)	5 (45.5)
Normal weight	49 (35.3)	27 (27.6)	28 (29.8)	38 (27.3)	21 (21.4)	24 (25.5)	93 (66.9)	59 (60.2)	68 (72.3)
Overweight	54 (28.0)	53 (28.8)	50 (26.6)	66 (34.2)	52 (28.3)	44 (23.4)	118 (61.1)	92 (50.0)	128 (68.1)
Obesity	39 (31.2)	41 (34.8)	36 (29.8)	30 (24.0)	26 (22.0)	37 (30.6)	80 (64.0)	58 (49.2)	79 (65.3)
Smoking status									
Smoker	33 (29.7)	42 (35.0)	32 (31.7)	16 (14.4)	13 (10.8)	12 (11.9)	85 (76.6)	87 (72.5)	76 (75.3)
Ex-smoker	79 (29.9)	56 (28.1)	68 (29.3)	92 (34.9)	62 (31.2)	70 (30.2)	154 (58.3)	85 (42.7)	160 (69.0)
Non-smoker	30 (36.6)	25 (28.7)	21 (25.9)	26 (31.7)	26 (29.9)	27 (33.3)	52 (63.4)	40 (46.0)	44 (54.3)
Alcohol intake in the last year									
Yes	18 (41.9)	12 (30.8)	63 (21.8)	19 (44.2)	4 (10.3)	58 (20.1)	27 (62.8)	14 (35.9)	197 (68.2)
No	124 (30.0)	111 (30.3)	58 (46.4)	115 (27.8)	97 (26.4)	51 (40.8)	264 (63.8)	198 (54.0)	83 (66.4)
Leisure-time physical activity									
Yes	74 (25.7)	41 (21.4)	51 (21.6)	74 (25.7)	27 (14.1)	36 (15.3)	172 (59.7)	87 (45.3)	158 (67.0)
No	68 (40.2)	82 (38.3)	70 (39.3)	60 (35.5)	74 (34.6)	73 (41.0)	119 (70.4)	125 (58.4)	122 (68.5)
Number of chronic conditions									
0	21 (26.3)	11 (18.0)	14 (18.7)	12 (15.0)	4 (6.6)	7 (9.3)	60 (75.0)	41 (67.2)	54 (72.0)
1–2	68 (26.9)	26 (22.8)	21 (20.0)	66 (26.1)	50 (22.4)	50 (21.6)	159 (62.9)	117 (52.5)	158 (68.1)
≥3	53 (42.7)	26 (23.9)	41 (32.3)	56 (45.2) ^1^	47 (38.5)	52 (48.6)	72 (58.1)	54 (44.3)	68 (63.6)
Measurement of blood pressure in the last 12 months									
Yes	131 (31.3)	112 (30.5)	114 (29.9)	127 (30.3)	95 (25.9)	107 (28.1)	259 (61.8)	188 (51.2)	253 (66.4)
No	11 (29.0)	11 (28.2)	7 (21.2)	7 (18.4)	6 (15.4)	2 (6.1)	32 (84.2)	24 (61.5)	27 (81.8)
Measurement of the amount of total serum cholesterol in the last 12 months									
Yes	117 (31.5)	102 (28.9)	109 (29.5)	109 (29.3)	93 (26.4)	106 (28.7)	234 (62.9)	179 (50.7)	245 (66.2)
No	25 (29.4)	21 (39.6)	12 (27.3)	25 (29.4)	8 (15.1)	3 (6.8)	57 (67.1)	33 (62.3)	35 (79.6)
Influenza vaccination during the last campaign									
Yes	101 (33.9)	67 (27.7)	66 (26.8)	108 (36.2)	73 (30.2)	77 (31.3)	169 (56.7)	114 (47.1)	150 (61.0)
No	41 (25.8)	56 (34.2)	55 (32.7)	26 (16.4)	28 (17.1)	32 (19.1)	122 (76.7)	98 (59.8)	130 (77.4)

PT: professional training; Inh: Inhabitants; ^1^ *p* for trend <0.001.

**Table 3:** Distribution of CMDs, functional limitation and poor/improvable diet quality according to the study variables in women with COPD from 2006 to 2017 (*n* = 1295).

	Common Mental Disorders			Functional Limitation			Poor/Improvable Diet Quality		
	*n* = 589			*n* = 381			*n* = 687		
**Variables**	2006	2011	2017	2006	2011	2017	2006	2011	2017
	*n* = 217 (%)	*n* = 182 (%)	*n* = 190 (%)	*n* = 114 (%)	*n* = 108 (%)	*n* = 159 (%)	*n* = 260 (%)	*n* = 166 (%)	*n* = 261 (%)
Age (years)									
40–54	57 (46.3)	41 (52.6)	37 (51.4)	-	-	-	92 (74.8)	47 (60.2)	54 (75.0)
55–69	74 (44.3)	72 (48.3)	54 (33.8)	20 (12.0)	16 (10.7)	20 (12.5)	81 (48.5)	60 (40.2)	97 (60.6)
≥70	86 (49.7)	69 (44.0)	99 (45.8)	94 (54.3)	92 (58.6)	139 (64.4)	87 (50.3)	59 (37.6)	110 (50.9)
Educational attainment									
Without studies	62 (48.4)	67 (52.3)	67 (51.2)	53 (41.4)	60 (46.9)	85 (64.9)	72 (56.3)	59 (46.1)	76 (58.0)
Primary	105 (48.6)	31 (50.0)	52 (42.6)	48 (22.2)	21 (33.9)	44 (36.1)	117 (54.2)	27 (43.6)	65 (53.3)
Secondary or PT	37 (44.6)	78 (46.2)	57 (36.1)	9 (10.8)	26 (15.4)	24 (15.2)	51 (61.5)	69 (40.8)	101 (63.9)
University	13 (36.1)	6 (24.0)	14 (37.8)	4 (11.1)	1 (4.0)	6 (16.2)	20 (55.6) ^1^	11 (44.0)	19 (51.4)
Town population									
<10,000 inh	53 (45.0)	44 (49.9)	35 (36.1)	31 (26.3)	28 (31.1)	39 (40.2)	70 (59.3)	33 (36.7)	57 (58.8)
10,000–100,000 inh	77 (45.0)	63 (53.4)	66 (44.9)	37 (21.6)	33 (28.0)	57 (38.8)	91 (53.2)	64 (54.2)	80 (54.4)
>100,000 inh	87 (50.0)	75 (42.6)	89 (43.6)	46 (26.4)	47 (26.7)	63 (30.1)	99 (56.9)	69 (39.2)	124 (60.8)
Living with a partner									
Yes	112 (45.0)	42 (46.2)	47 (37.6)	41 (16.5)	21 (23.1)	37 (29.6)	138 (55.4)	38 (41.8)	76 (60.8)
No	105 (49.1)	140 (47.8)	143 (44.2)	73 (34.1)	87 (29.7)	122 (37.8)	122 (57.0)	128 (43.7)	185 (57.3)
Occupation									
Working	26 (30.6)	19 (30.2)	19 (31.2)	1 (1.2)	-	1 (1.6)	54 (63.5)	34 (54.0)	46 (75.4)
Non-working	191 (50.5)	163 (50.8)	171 (44.2)	113 (29.9)	108 (33.6)	158 (40.8)	206 (54.5)	132 (41.1)	215 (55.6)
Body Mass Index									
Underweight	4 (50.0)	3 (60.0)	3 (75.0)	3 (37.5)	-	2 (50.0)	8 (100.0)	3 (60.0)	3 (75.0)
Normal weight	61 (41.8)	33 (32.7)	61 (43.0)	29 (19.9)	21 (20.8)	48 (33.8)	88 (60.3)	46 (45.5)	81 (57.0)
Overweight	77 (45.6)	63 (52.5)	59 (40.4)	34 (20.1)	29 (24.2)	45 (30.8)	93 (55.0)	51 (42.5)	84 (57.5)
Obesity	75 (53.6)	83 (52.5)	67 (43.0)	48 (34.3)^1^	58 (36.7)	64 (41.0)	71 (50.7)	66 (41.8)	93 (59.6)
Smoking status									
Smoker	40 (43.0)	46 (59.7)	35 (46.1)	4 (4.3)	3 (3.9)	7 (9.2)	65 (69.9)	54 (70.1)	55 (72.4)
Ex-smoker	26 (53.1)	18 (32.7)	30 (33.7)	4 (8.2)	7 (12.7)	19 (21.4)	28 (57.1)	17 (100.0)	51 (57.3)
Non-smoker	151 (47.0)	118 (46.8)	125 (44.2)	106 (33.0)	98 (38.9)	133 (47.0)	167 (52.0)	95 (37.7)	155 (54.8)
Alcohol intake in the last year									
Yes	27 (48.2)	20 (41.7)	63 (31.3)	6 (10.7)	6 (12.5)	36 (17.9)	36 (64.3)	25 (52.1)	117 (58.2)
No	190 (46.7)	162 (48.2)	127 (51.4)	108 (26.5)	102 (30.4)	123 (49.8)	224 (55.0)	141 (42.0)	144 (58.3)
Leisure-time physical activity									
Yes	103 (39.9)	65 (36.3)	71 (33.4)	44 (17.1)	22 (12.3)	39 (18.5)	128 (49.6)	71 (39.7)	128 (60.7)
No	114 (55.6)	117 (57.1)	119 (50.2)	70 (34.2)	86 (42.0)	120 (50.6)	132 (64.4)	95 (46.3)	133 (56.1)
Number of chronic conditions									
0	10 (17.9)	19 (35.9)	14 (33.3)	2 (3.3)	4 (7.6)	5 (11.9)	39 (69.6)	35 (66.0)	31 (73.8)
1–2	114 (44.4)	69 (37.1)	88 (37.1)	53 (20.6)	32 (17.2)	57 (24.1)	154 (59.9)	75 (40.3)	134 (56.5)
≥3	93 (62.0)	94 (64.8)	88 (52.1)	59 (39.3)^1^	72 (49.7)	97 (57.4)	67 (44.7)	56 (38.6)	96 (56.8)
Measurement of blood pressure in the last 12 months									
Yes	203 (48.9)	174 (49.3)	177 (43.2)	110 (26.5)	107 (30.3)	156 (38.1)	224 (54.0)	149 (42.2)	238 (58.1)
No	14 (29.2)	8 (25.8)	13 (34.2)	4 (8.3)	1 (3.2)	3 (7.9)	36 (75.0)	17 (54.8)	23 (60.5)
Measurement of the amount of total serum cholesterol in the last 12 months									
Yes	190 (49.4)	164 (50.3)	171 (43.0)	100 (26.0)	98 (30.1)	154 (38.7)	202 (52.5)	132 (40.5)	223 (56.0)
No	27 (34.6)	18 (31.0)	19 (38.0)	14 (18.0)	10 (17.2)	5 (10.0)	58 (74.4)	34 (58.6)	38 (76.0)
Influenza vaccination during the last vaccination campaign									
Yes	118 (45.4)	86 (43.7)	99 (41.6)	90 (34.6)	64 (32.5)	115 (48.3)	143 (55.0)	62 (31.5)	129 (54.2)
No	99 (48.8)	96 (51.3)	91 (43.3)	24 (11.8)	44 (23.5)	44 (21.0)	117 (57.6)	104 (55.6)	132 (62.9)

PT: professional training; Inh: Inhabitants; ^1^ *p* for trend < 0.001.

**Table 4:** Association between CMDs, functional limitation and poor/improvable diet quality, and sociodemographic factors, health-related determinants and use of clinical preventive care services in men with COPD (*n* = 1277).

Variables	Common Mental Disorders			Functional Limitation			Poor/Improvable Diet Quality		
	OR (IC 95%)	ORa (IC95%)	*p*-Value	OR (IC 95%)	ORa (IC95%)	*p*-Value	OR (IC 95%)	ORa (IC95%)	*p*-Value
Age group									
40–54	1.11 (0.80–1.55)			-			2..93 (2.03–4.24)	1.94 (1.28–2.96)	0.01
55–69	0.82 (0.63–1.08)			0.13 (0.09–0.18)			1.21 (0.94–1.54)	0.98 (0.75–1.29)	0.09
≥70	Reference			Reference			Reference	Reference	
Educational attainment									
Without studies	Reference	Reference		Reference	Reference		Reference		
Primary	0.79 (0.58–1.08)	0.89 (0.64–1.24)	0.49	0.59 (0.43–0.80)	0.63 (0.46–0.87)	<0.01	1.38 (0.34–1.85)		
Secondary	0.83 (0.61–1.13)	1.02 (0.73–1.41)	0.62	0.23 (0.16–0.32)	0.25 (0.17–0.36)	<0.001	1.60 (0.19–2.16)		
University	0.44 (0.26–0.74)	0.55 (0.32–0.96)	0.03	0.40 (0.24–0.66)	0.51 (0.30–0.86)	0.01	1.19 (0.77–1.84)		
Town population (inhabitants)									
<10,000	0.96 (0.71–1.30)			1.27 (0.93–1.72)			1016 (0.88–1.53)		
10,000–100,000	1.41 (1.06–1.88)			1.35 (0.99–1.81)			0.92 (0.70–1.20)		
>100,000	Reference			Reference			Reference		
Living with a partner									
Yes	0.72 (0.57–0.91)			1.10 (0.85–1.40)			0.72 (0.57–0.90)		
No	Reference			Reference			Reference		
Occupation									
Working	0.68 (0.48–0.96)			-			2.28 (1.63–3.20)		
Non-working	Reference			Reference			Reference		
BMI									
Underweight	2.92 (1.11–7.71)	2.42 (0.88–6.63)	0.09	1.36 (0.49–3.73)			0.60 (0.23–1.57)		
Normal	0.19 (0.08–0.60)	0.37 (0.10–0.88)	0.04	0.83 (0.61–1.13)			1.33 (1.01–1.76)		
Overweight	Reference	Reference		Reference			Reference		
Obesity	1.22 (0.91–1.62)	1.06 (0.78–1.44)	0.69	0.85 (0.63–1.15)			0.99 (0.76–1.30)		
Smoking status									
Smoker	1.15 (0.87–1.53)			0.30 (0.21–0.43)			2.19 (1.64–2.93)	1.67 (1.22–2.29)	<0.01
Ex-smoker	Reference			Reference			Reference	Reference	
Non-smoker	1.06 (0.77–1.45)			0.97 (0.71–1.33)			0.89 (0.66–1.18)	0.80 (0.59–1.08)	0.13
Alcohol intake ^1^									
Yes	1.70 (1.53–1.92)	1.73 (1.55–1.97)	0.02	0.68 (0.51–0.91)			1.19 (0.92–1.52)		
No	Reference	Reference		Reference			Reference		
Leisure-time physical activity									
Yes	0.47 (0.37–0.60)	0.51 (0.40–0.66)	<0.001	0.41 (0.31–0.52)	0.43 (0.33–0.56)	<0.001	0.74 (0.59–0.93)	0.76 (0.60–0.97)	0.03
No	Reference	Reference		Reference	Reference		Reference	Reference	
Number of chronic conditions									
0	0.33 (0.23–0.49)	0.33 (0.22–0.50)	<0.001	0.15 (0.09–0.25)	0.18 (0.11–0.30)	<0.001	2.08 (1.45–2.99)		
1–2	0.43 (0.33–0.56)	0.44 (0.34–0.59)	<0.001	0.39 (0.30–0.51)	0.45 (0.33–0.59)	<0.001	1.30 (1.01–1.69)		
≥3	Reference	Reference		Reference	Reference		Reference		
Measurement of blood pressure ^1^									
Yes	Reference			Reference			Reference		
No	0.81 (0.52–1.27)			0.40 (0.23–0.70)			2.05 (0.71–3.22)		
Measurement of the amount of total serum cholesterol ^1^									
Yes	Reference			Reference			Reference		
No	1.09 (0.78–1.53)			0.63 (0.43–0.93)			1.46 (0.84–2.04)		
Influenza vaccination during the last campaign									
Yes	Reference			Reference			Reference		
No	1.06 (0.83–1.35)			0.44 (0.33–0.57)			2.02 (0.59–2.58)		

^1^ in the last 12 months; BMI: body mass index; PT: professional training; OR: odds ratio; ORa: odds ratio adjusted for all sociodemographic variables, health-related determinants and use of clinical preventive care services; CI 95%: 95% Confidence Interval; Hosmer-Lemeshow test for CMDs χ^2^ = 4.16, *p* = 0.84; Nagelkerke’s R^2^ Square for CMD = 0.11; *p*-value < 0.001. Hosmer-Lemeshow test for functional limitation χ^2^ = 7.32, *p* = 0.40; Nagelkerke’s R^2^ Square for functional limitation = 0.20; *p*-value < 0.001. Hosmer-Lemeshow test for poor/improvable diet quality χ^2^ = 6.48, *p* = 0.59; Nagelkerke’s R^2^ Square for poor/improvable diet quality = 0.09; *p*-value < 0.001.

**Table 5:** Association between CMDs, functional limitation and poor/improvable diet quality and sociodemographic factors, health-related determinants and use of clinical preventive care services in women with COPD (*n* = 1295).

Variables	Common Mental Disorders			Functional Limitation			Poor/Improvable Diet Quality		
	OR (IC 95%)	ORa (IC95%)	*p*-Value	OR (IC 95%)	ORa (IC 95%)	*p*-Value	OR (IC 95%)	ORa (IC 95%)	*p*-Value
Age group									
40–54	1.13 (0.84–1.50)			-	-		2.73 (2.01–3.73)	2.02 (1.43–2.87)	<0.001
55–69	0.83 (0.65–1.07)			0.91 (0.07–0.13)	0.14 (0.10–0.20)	<0.001	1.13 (0.89–1.45)	0.99 (0.76–1.29)	0.94
≥70	Reference			Reference	Reference		Reference	Reference	
Educational attainment									
Without studies	Reference			Reference	Reference		Reference		
Primary	0.86 (0.65–1.14)			0.38 (0.28–0.51)	0.70 (0.48–0.85)	0.04	0.95 (0.72–1.26)		
Secondary	0.70 (0.53–0.93)			0.16 (0.11–0.23)	0.55 (0.36–0.84)	<0.01	1.02 (0.77–1.34)		
University	0.50 (0.31–0.79)			0.12 (0.06–0.23)	0.69 (0.30–0.93)	0.04	0.91 (0.58–1.41)		
Town population (inhabitants)									
<10,000	0.92 (0.70–1.22)			1.21 (0.89–1.64)			0.99 (0.79–1.31)		
10,000–100,000	1.08 (0.84–1.39)			1.05 (0.80–1.38)			1.05 (0.82–1.35)		
>100,000	Reference			Reference			Reference		
Living with a partner									
Yes	0.87 (0.69–1.09)			0.53 (0.40–0.69)	0.67 (0.48–0.95)	<0.001	1.07 (0.86–1.35)		
No	Reference			Reference	Reference		Reference		
Occupation									
Working	0.47 (0.34–0.65)	0.61 (0.43–0.86)	<0.01	0.02 (0.01–0.07)			1.72 (1.27–2.34)		
Non-working	Reference	Reference		Reference			Reference		
BMI									
Underweight	1.69 (0.63–4.53)			1.26 (0.44–3.66)			4.24 (1.20–1.49)	4.06 (1.12–4.65)	0.03
Normal	0.79 (0.60–1.04)			1.02 (0.74–1.40)			1.12 (0.85–1.48)	0.96 (0.72–1.28)	0.80
Overweight	Reference			Reference			Reference	Reference	
Obesity	1.17 (0.90–1.52)			1.81 (1.36–2.42)			0.93 (0.72–1.21)	0.99 (0.76–1.31)	0.98
Smoking status									
Smoker	1.56 (1.06–2.28)	2.01 (1.34–3.01)	<0.01	0.34 (0.17–0.64)			2.44 (1.65–3.62)	2.02 (1.35–3.04)	<0.01
Ex-smoker	Reference	Reference		Reference			Reference	Reference	0.51
Non-smoker	1.37 (0.99–1.89)	1.04 (0.74–1.46)	0.81	3.53 (2.34–5.33)			0.96 (0.70–1.31)	1.12 (0.80–1.55)	
Alcohol intake ^1^									
Yes	1.60 (1.46–1.78)	1.69 (1.51–1.88)	<0.01	0.37 (0.26–0.52)			1.32 (1.02–1.72)		
No	Reference	Reference		Reference			Reference		
Leisure-time physical activity									
Yes	0.50 (0.40–1.62)			0.26 (0.20–0.34)	0.30 (0.22–0.41)	<0.001	0.81 (0.65–1.01)		
No	Reference			Reference	Reference		Reference		
Number of chronic conditions									
0	0.27 (0.18–0.41)	0.30 (0.19–0.46)	<0.001	0.08 (0.04–0.15)	0.43 (0.19–0.95)	0.04	2.55 (1.73–3.78)		
1–2	0.46 (0.36–0.58)	0.48 (0.37–0.62)	<0.001	0.27 (0.21–0.35)	0.48 (0.35–0.67)	<0.001	1.28 (1.01–1.62)		
≥3	Reference	Reference		Reference	Reference		Reference		
Measurement of blood pressure ^1^									
Yes	Reference			Reference			Reference		
No	0.48 (0.32–0.73)			0.16 (0.08–0.33)			1.72 (0.16–2.56)		
Measurement of the amount of total serum cholesterol ^1^									
Yes	Reference			Reference			Reference		
No	0.58 (0.42–1.81)			0.40 (0.26–1.60)			2.30 (0.65–3.22)		
Influenza vaccination during the last campaign									
Yes	Reference			Reference			Reference		
No	1.18 (0.95–1.47)			0.36 (0.80–1.47)			1.55 (0.24–1.93)		

^1^ in the last 12 months; BMI: body mass index; PT: professional training; OR: odds ratio; ORa: odds ratio adjusted for all sociodemographic variables, health-related determinants and use of clinical preventive care services; CI 95%: 95% Confidence Interval; Hosmer-Lemeshow test for CMDs χ^2^ = 6.51, *p* = 0.37; Nagelkerke’s R^2^ Square for CMD = 0.10; *p*-value < 0.001. Hosmer-Lemeshow test for functional limitation χ^2^ = 7.50, *p* = 0.48; Nagelkerke’s R^2^ Square for functional limitation = 0.54; *p*-value < 0.001. Hosmer-Lemeshow test for poor/improvable diet quality χ^2^ = 6.22, *p* = 0.62; Nagelkerke’s R^2^ Square for poor/improvable diet quality = 0.08; *p*-value < 0.001.

## Data Availability

The data presented in this study are available as Supplementary Material (File S1: Research data).

## Supplementary Materials

The following are available online at https://www.mdpi.com/article/10.3390/jcm10112291/s1, Table S1: Criteria to define the score for each item of the Spanish Health Eating Index (SHEI), File S1: Research data.
