# Supplementary material for: Common Mental Disorders, Functional Limitation and Diet Quality Trends and Related Factors among COPD Patients in Spain, 2006–2017: Evidence from Spanish National Health Surveys
Source: J Clin Med. 2021 May 25;10(11):2291. doi: 10.3390/jcm10112291 (PMC8197509; doi:10.3390/jcm10112291)
Supplement: Supplementary file 1 [file jcm-10-02291-s001.zip › Supplementary Table S1.pdf]

Supplementary Table S1. Criteria to define the score for each item of the Spanish Health Eating Index (SHEI).

Criteria to define the score for each item of the Spanish Health Eating Index (SHEI)					
**Variables**	Criteria for a maximum score of 10	Criteria for a maximum score of 7.5	Criteria for a maximum score of 5	Criteria for a maximum score of 2.5	Criteria for a maximum score of 0
Daily					
Bread or grains	Daily	Three or more times a week, but not daily	Once or twice a week	Less than once a week	Never or almost never
Leafy greens, salads and vegetables	Daily	Three or more times a week, but not daily	Once or twice a week	Less than once a week	Never or almost never
Fresh fruit (excluding juices)	Daily	Three or more times a week, but not daily	Once or twice a week	Less than once a week	Never or almost never
Dairy products (milk, cheese, yoghurt)	Daily	Three or more times a week, but not daily	Once or twice a week	Less than once a week	Never or almost never
Weekly consumption					
Meat (chicken, beef, pork, lamb, etc.)	Once or twice a week	Three or more times a week, but not daily	Less than once a week	Daily	Never or almost never
Legumes	Once or twice a week	Three or more times a week, but not daily	Less than once a week	Daily	Never or almost never
Occasional consumption					
Cold meats and cuts	Never or almost never	Less than once a week	Once or twice a week	Three or more times a week, but not daily	Daily
Sweets (biscuits, pastries, jams, cereals with sugar, sweets, etc.)	Never or almost never	Less than once a week	Once or twice a week	Three or more times a week, but not daily	Daily
Soft drinks with sugar	Never or almost never	Less than once a week	Once or twice a week	Three or more times a week, but not daily	Daily
Variety	2 points if participant achieve each of the daily recommendations, 1 point if participant achieve each of the weekly recommendations.				

Each item scored from 0 to 10 depending to the criteria of the Spanish Health Eating Index (SHEI) [1], which 10 points means that the recommendations proposed by the Spanish Society of Community Nutrition (SSCN) [2].

Supplementary references

1. Norte Navarro, A.; Ortiz Moncada, R. Spanish diet quality according to the healthy eating index. *Nutr. Hosp.* **2011**, *26*, 330–336, doi: 10.1590/S0212-16112011000200014.
2. Spanish Society of Community Nutrition (SSCN). Healthy dietary guidelines (2014). Available online: http://www.nutricioncomunitaria.org/es/otras-publicaciones (accessed on 13 December 2020).
